# Analysis of the implementation of a community-based intervention to control dengue fever in Burkina Faso

**DOI:** 10.1186/s13012-020-00989-x

**Published:** 2020-05-14

**Authors:** Issa Sombié, Stéphanie Degroote, Paul André Somé, Valéry Ridde

**Affiliations:** 1 Institut des Sciences des Sociétés /CNRST, 03 BP 7047, Ouagadougou, 03 Burkina Faso; 2 AGIR/SD (Action, Gouvernance, Intégration et Renforcement en Santé et Développement), 14 BP 254, Ouagadougou, 14 Burkina Faso; 3 Institut de Recherches pour le Développement (IRD), Centre Population et Développement (CEPED), Université Sorbonne Paris Cité, ERL INSERM SAGESUD, 45 rue des Saints-Pères, 75006 Paris, France; 4 University of Montreal Public Health Research Institute (IRSPUM), Montreal, Canada

**Keywords:** Analysis of implementation, CFIR, Community intervention, Dengue fever, Burkina Faso

## Abstract

**Background:**

A community-based dengue fever intervention was implemented in Burkina Faso in 2017. The results achieved vary from one area to another. The objective of this article is to analyze the implementation of this intervention, to better understand the process, and to explain the contextual elements of performance variations in implementation.

**Methodology:**

The research was conducted in the former sector 22 of the city of Ouagadougou. We adapted the Consolidated Framework for Implementation Research (CFIR) to take into account the realities of the context and the intervention. The data collected from the participants directly involved in the implementation using three techniques: document consultation, individual interview, and focus group.

**Results:**

Two dimensions of CFIR emerge from the results as having had a positive influence on the implementation: (i) the characteristics of the intervention and (ii) the processes of the intervention implementation. The majority of the CFIR constructions were considered to have had a positive effect on implementation. The quality and strength of the evidence received the highest score. The dimension of the external context had a negative influence on the implementation of the intervention.

**Conclusion:**

The objective of the study was to analyze the influence of contextual elements on the implementation process of a community-based dengue fever intervention. We used the CFIR framework already used by many studies for implementation analysis. Although it was not possible to test this framework in its entirety, it is useful for the analysis of the implementation. Its use is simple and does not require any special skills from users. Usability is indeed an essential criterion for the relevance of using an analytical framework in implementation science.

Contribution to the literature

- This study shows that when an intervention is designed taking into account the expectations of the main participants, and the realities of the local context, its implementation benefits from a sincere commitment from the beneficiaries.
- The application of the CFIR framework and its five dimensions inevitably requires its contextualization, especially when it is used to analyze interventions that are not patient-oriented and in a context different from health facilities.
- The CFIR framework is a relevant analytical tool in the West African context where multiple interventions are implemented at the same time and some fail to achieve their objectives.

## Background

Dengue fever is a little-known disease that is very often confused with malaria. It is a viral infection that is prevalent in many Latin American, Asian, and African countries. The incidence of this disease has increased worldwide in recent decades [1], but the situation varies greatly from continent to continent and country to country. Thus, it is important to identify interventions that are adaptable to different contexts. Thus, the effectiveness of many dengue-control initiatives (community-based environmental management for malaria control, community-based control of *Aedes aegypti*) implemented by communities in several countries in Asia and Latin America have already been demonstrated [2–5]. In many cases, those seeking to learn from these strategies do not have information on what is essential for their successful implementation [6]. However, there is little research that explains the reasons for this and reports on its implementation [7]. This article aims to present and explain the elements that have contributed to the success of the community-based intervention to fight dengue fever in Burkina Faso. These elements can be used as a guide for other similar interventions elsewhere.

There is a growing body of research that attempts to understand the role of context in the implementation of interventions [6], even if the concept of context is still new. Several recent systematic reviews have been published on the role of context in interventions [8, 9]. A group of researchers has recently proposed a number of questions for researchers, implementors, funders, and scientific journals to further explore the role of context [9–11]. However, such research is rarer in Africa than in other continents, as is also the case for process analyses [12]. A realistic review of health care payment exemption policies in Africa has thus made it possible to highlight the importance of the role of context in explaining their effectiveness and implementation [12, 13]. In Burkina Faso, the context of this article, we have also shown how the context influences the effectiveness of a maternal health policy centered on payment exemption [13].

In Burkina Faso, located in West Africa, dengue fever has only been registered in the epidemiological surveillance system of the Ministry of Health since 2014. However, the first cases of dengue fever were reported in 1925, and many cases were detected in 1985 [14, 15]. In 2003, a study of 191 blood donors and 492 pregnant women in two districts indicated that at least 26% had had contact with one of the viruses that transmit dengue fever [16]. In 2013, hospital services in Ouagadougou reported 33 positive cases of dengue fever and diagnosed 18 cases of dengue fever on a sample of 264 children [17].

It is in this context that a community intervention to combat dengue fever has been set up in the city of Ouagadougou and more precisely around the health facility in sector 22. The main activities developed are awareness and information campaigns, small group discussions, the installation of several posters, theater sessions, and demonstration sessions on the identification of breeding sites and their destruction. Approximately 3000 people were reached by the outreach activities during the 6 months of the intervention.

This community-based intervention to control dengue fever in Burkina Faso [18] implemented using a participatory approach in June 2016 was structured around several components: the use of a local non-governmental organization (NGO) to implement the intervention, the selection of activities based on evidence and validated by the populations concerned, the identification of community preferences for these activities, the NGO’s support for community mobilization and engagement, the development of communication materials and the training of community facilitators to support the improvement of the population’s knowledge and skills, and the organization of communities and their support in monitoring activities. Three groups of participants participated in the implementation of the activities: the facilitators appointed by the main health promotion association active in each of the sub-areas and trained by the NGO Association Action Gouvernance, Intégration et Renforcement (AGIR/SD is a local association working in the field of health promotion. It is the association that has implemented, in collaboration with other actors, the community-based intervention in the control of dengue fever in Burkina Faso), the members of the monitoring committee, and the project team within AGIR/SD. A study conducted on the implementation fidelity of this intervention [19] concluded that it was implemented as designed throughout the study area. A second study [20] on the effectiveness of this intervention showed that it had achieved the expected results of improving knowledge and reducing the risk of exposure to dengue fever among the populations in the study area. However, the researchers reported that there was a variation in the results according to the different sub-areas of intervention. The objective of this article is to report on the evaluation of the process of intervention implementation. This type of evaluation is important because it helps to explain variations in performance between different areas of intervention.

## Methods

### Design

The study design is a population health intervention research [21] that uses the single-case study methodological strategy [22]. This type of design is recommended when studying the implementation of a complex intervention in its natural context where researchers have no control over the elements that can influence it [23]. The case is the community intervention implemented in Ouagadougou. The different levels of analysis are linked to the dimensions of the analytical framework presented below.

### Context

The study took place between December 2016 and April 2017 in Burkina Faso’s capital city, Ouagadougou. The city is subdivided into 12 districts and has a population of about two million. The healthcare offered is organised into four health districts. The intervention was implemented in the Tampouy Health district and more precisely in a specific administrative sector. In Burkina Faso, an administrative sector is an area covering approximately a population of more than 300 people. The project activities reached areas within a radius of 1 km around the health center because they were associated with a seroprevalence study [24]. The activities were implemented in three sub-areas: Yitouni, Cité Azimo & AnIV B, and Tampouy Bilbalogho. Ethics approval to conduct the study was given by the National Health Ethics Committee.

### Conceptual framework

The implementation analysis is based on the use of the Consolidated Framework for Implementation Research (CFIR) [25]. The CFIR is a conceptual meta-framework that synthesizes analytical elements for the study of the implementation of an intervention from several frameworks borrowed from psychology, sociology, and administrative science [26, 27]. It consists of five dimensions (Table 1) that include several constructs to address various aspects related to the implementation of an intervention. As recommended by the authors [25], CFIR was adapted to take into account the context of the intervention. Two criteria were taken into account when choosing the framework constructs to be used, namely the nature of this community intervention and the availability of data. The CFIR has been used extensively for the analysis of patient interventions at the health service level [28]. However, since the intervention is a community and population-based intervention, some elements of the framework were difficult to apply (e.g., patient needs and resources, peer pressure, organizational incentives, and rewards). The absence of empirical data for some constructs (i.e., relative advantage) did not allow them to be retained. For others, we do not have sufficient data to analyze them. Thus, Table 1 presents the dimensions used for the analysis as well as the constructs used and their description.

**Table 1:** Dimensions and constructs of the analytical framework and their description

Dimensions	Constructs	Description
1. Characteristics of the intervention	1.2 Origin of the intervention	Stakeholders' perception of the origin of the intervention.
	1.2 Quality and Strength of Evidence	Stakeholders' perceptions of the quality and validity of evidence that the intervention will achieve its intended outcomes (i.e. intervention theory).
	1.3 Adaptability	The degree to which the intervention can be adapted or reinvented to meet local needs.
	1.4 Complexity	The perceived difficulty of implementing the intervention, particularly in terms of duration, scope, level of disruption, centrality, and complexity, and the number of steps required to implement it.
2. External Context	2.1 Network	The degree to which the organization implementing the intervention is networked with external organizations.
3. Internal Context	3.1 Structural characteristics	The social architecture, age, maturity, and size of the organization implementing the intervention.
	3.2 Networks and communications	The nature and quality of social networks and formal and informal communications in the organization.
	3.3 Preparation of implementation	Tangible and immediate indicators of organizational commitment to implement the intervention.
	3.4 Commitment of the leaders	The commitment, involvement, and responsibility of the leaders and managers of AGIR (the organization that implements the intervention) with regard to implementation.
	3.5 Available resources	The amount of resources devoted to implementation and operations, including money, training, and education, physical space, and time.
4. Characteristics of individuals	4.1 Knowledge and beliefs about the intervention	Individual attitudes towards the intervention and the values attributed to the intervention, as well as knowledge of the facts, truths, and principles related to the intervention.
	4.2 Self-efficacy	Individuals' belief in their own ability to execute action plans to achieve implementation goals.
5. Process	5.1 Planification	The degree to which plans, methods, and tasks for implementing an intervention are developed in advance and the quality of these methods.
	5.2 Implication	Attract and involve appropriate people in the implementation and use of the intervention through a combined strategy of social marketing, education, role modeling, training, and other similar activities.
	*5.3* Formally appointed internal leaders for implementation	The members of the association promoting the intervention who have distinguished themselves by their dedication and commitment to implementation.
	*5.4* Champions	People who are dedicated to supporting, marketing, and conducting the implementation, and to overcoming the indifference or resistance that the intervention can cause in an organization.

*Source: Adapted from Damschroder et al.* [28]

### Study population

The study population consists of people who have been involved in the implementation of the intervention activities. These people were grouped into three groups: the facilitators, the members of the monitoring committee, and the members of the association AGIR. The facilitators are men and women who have been chosen from among the members of the associations working in the area to participate in the implementation of the activities. Based on their experience and availability, they were chosen to join the project’s field team. The monitoring committee is a body composed of religious and customary leaders, heads of associations to support the implementation of project activities. Its main role was to facilitate the implementation of activities through public awareness and technical support to facilitators. Only one criterion was used to select participants: to be a member of the intervention implementation team. The objectives of the study were explained to them beforehand. Coming from several associations working in the health area of specific administrative sectors, the facilitators form a group of 17 peoples including 16 women and 1 man. Their ages range from 20 to 59 years and the average age is 42 years. The majority of them have not completed primary school. They are the ones who organized and carried out the household’s talk and door-to-door activities. With regard to the members of the Monitoring Committee, their ages range from 39 to 69 years, and the average age is 56 years. Only one woman is in the group. The project team (three people) of the association AGIR and an intern participated in the implementation of the intervention activities through their presence, their involvement in planning and practical organization, and follow-up.

### Data collection

Data collection took place in December 2016 using three methods: document consultation, focus group discussion, and individual interviews. Information was extracted from activity reports, supervision reports, and management documents. Three focus group discussions were held with the facilitators (*n* = 17), members of the monitoring committee (*n* = 7), and members of the AGIR association and its trainees (*n* = 5). The focus group interviews with the facilitators and members of the monitoring committee were conducted in Mooré language and facilitated by two trainees and one member of AGIR. The focus group discussion with AGIR members was facilitated by the first author in French. The focus group discussion and interview guides were developed based on the elements of the CFIR framework selected (Table 1). A scoring grid (Table 2) was designed based on the example of Damschroder and colleagues [25]. It was used to assess the influence of each element of the framework on the intervention. This grid was not used to collect quantitative data but simply as a tool to facilitate collective consensus among stakeholders around the different points discussed and thus prepare qualitative data collection guides. The first author (IS) explained the overall approach and the content of the scoring grid. Within each subgroup, participants discussed the questions proposed by the framework before indicating, through the rating given, whether the factor in question had a negative, positive, or no impact on the intervention (Table 2). The scores, ranging from − 2 to 2, were assigned by consensus. In addition to the group discussions, individual interviews were conducted with three facilitators and two members of the monitoring committee. The topics covered included the conditions for setting up the monitoring committee, its functioning, the relationships between its members and the facilitators, the understanding of the missions of this body, the contribution of association leaders, the relationships between the facilitators, the lessons learned, and the perspectives.

**Table 2:** Scoring grid for constructs

Note	Criteria/explanation
-2	The dimension had a negative influence on the implementation process. Participants were able to present concrete examples of negative influence.
-1	The dimension had a negative influence on the implementation process. Participants were unable to provide concrete examples to explain this influence.
0	Participants cannot expect the effective nature of the influence. While some believe that it has had a negative influence, others argue otherwise.
1	The dimension had a positive influence on the process by facilitating some aspects of implementation. Participants were unable to present facts that support their statements.
2	The dimension had a positive influence on the process by facilitating certain aspects of implementation. Participants presented facts that support their statements.

Source: Adapted from Damschroder et al. [28]

### Data processing and analysis

The data analysis was based on the framework analysis approach [29]. The scoring and comment grids were created using Microsoft Excel and Word software. This consisted of entering data from each sub-area into each grid separately. Then, a summary table containing data from all the three sub-areas in which the data were collected was designed to serve as a basis for analysis. The evaluated constructs were placed in columns and notes in rows to facilitate a comparison between the three sub-areas. The interviews were recorded and listened to several times to extract verbatim quotes, which were used to support and illustrate the analyses that were carried out with regard to the dimensions of the CFIR. The use of scores is only intended to provide an overall view of participants’ perceptions and to explore details through a qualitative approach, so they have no quantitative or statistical significance.

## Results

Table 3 summarizes the scores assigned by the study participants to constructs of the framework used. Responses vary very little from one sub-area to another. Qualitative results are presented according to the five dimensions of the CFIR. When there are specificities to a particular area, they are explained in the results or they have been shared between the three areas.

**Table 3:** Summary of consensus ratings for the various elements of the analytical framework

Constructs of the conceptual framework	Intervention areas		
	Yitouni	Cité Azimo & AnIV B	Tampouy Bilbalogo
1. Characteristics of intervention			
Origin of intervention	2	2	1
Quality and Strength of Evidence	2	2	2
Adaptability	1	1	− 1
Complexity	2	2	1
2. External context			
Network	− 1	− 2	− 2
3. Internal context			
Structural characteristics	− 2	1	− 2
Networks and communications	1	1	2
Preparation of implementation	1	1	0
Commitment of the leaders	1	2	2
Available resources	− 1	1	2
4. Characteristics of individuals			
Knowledge and beliefs about the intervention	1	2	2
Self-efficacy	2	− 1	1
5. Process			
Planification	2	1	2
Implication	1	2	1
Formally appointed internal leaders for implementation	1	2	2
*Champions*	2	0	− 1

### Characteristics of intervention

#### Origin of intervention

The project development process was considered participatory. This made it possible to take into account the expectations of all the participants involved in the implementation of the intervention. Participants indicated that the fact that their ideas were taken into account in the development of the intervention produced a sense of pride and satisfaction. This has helped to create a climate of engagement and frank collaboration among the various partners. The fact that the intervention was developed on the basis of proposals made by local participants facilitated their mobilization and involvement in the implementation of activities, as they acknowledged.

#### Quality and strength of evidence

Based on previous experiences in malaria control, which consisted mainly of mass spraying in neighborhoods, participants felt that the strategy used in this intervention was better. Indeed, the fact that the affected populations have raised awareness and trained in mosquito control techniques is more beneficial to them. The knowledge and skills they have acquired have made them more able to invest in promoting their health. From now on, they organize themselves in the neighborhoods to clean up their environment without waiting for the intervention of the political and administrative authorities.

*“You know, if you want to mobilize people around something, they have to understand it before they can get involved. It’s the same here. When the people in the neighbourhood had no information, they were not interested in the activities. But recently, that has changed. We organize at least once a month to clean the neighbourhood and plug up dirty water points that encourage mosquitoes to proliferate.” (Male, member of the monitoring committee).*

#### Adaptability

The adaptability of the intervention was not perceived in the same way at the level of the three sub-areas. While in the Yitouni and Cité Azimo areas, the content of the intervention corresponded to the needs of the populations; in the Tampouy Bilbalogho area, it was felt that the proposed activities did not reflect the expectations of the beneficiaries:

“I think this intervention has really met the needs of the people. It allowed them to get to know dengue fever and its prevention methods better. What else could we do but what was done. (Student, animator, Yitouni Zone)

During the talks, people thought that the most effective action would be a mass spray to kill mosquitoes. They thought that what we were doing was good, but not enough to protect them from mosquitoes. In any case, what many thoughts and did not think it was useful to take part in the activities”. (Facilitator, Tampouy Bilbalogho Zone).

Mobilization for the implementation of the intervention was influenced by the perceptions of participants.

Those who felt that project activities did not meet their expectations participated less than in the other two sub-areas.

#### Complexity

*“No, we can't say that this intervention was complex, everything was simple. Everything we did, almost all the facilitators have already done.”*

This sentence from a facilitator summarizes the general state of mind of the participants on the question of the complexity of the intervention. It is part of the global scheme of interventions promoted by local associations. The training received, the tools used, and the animation techniques are elements with which the field participants are familiar. They mentioned that the tools used were easy to understand and inform, the content and facilitation of the training corresponded to their level of education, the timeline of activities was fluid and the activities were synchronized. This perception of the simplicity of the intervention was a factor in the mobilization and motivation of the various participants involved in the implementation.

*“If you can perform the tasks entrusted to you properly and without difficulty, it encourages you to get more involved.” (Facilitator, Yitouni area)*

## External context

### Network

AGIR/SD, who is the operator of the intervention, were not operating in the intervention zone prior to this project. Therefore, it was not possible to have the contribution of any network. Although AGIR has been active in the city of Ouagadougou for several years, its scope of intervention had not yet reached the health area of a specific administrative sector. This explains why it has not been able to develop relations with the state, private, and associative structures in the area. The poor involvement of the Association AGIR in the networks at the level of the intervention area did not facilitate the implementation of certain activities, as one manager noted:

*“It must be acknowledged that our association was unable to establish solid relationships with community organizations in the area before the intervention began, which constituted a handicap. For example, there are two very active associations in the area that did not participate in the implementation of the intervention. Their experiences and networks of relationships could improve the performance of the dengue fever intervention” (AGIR Member, Man)*

## Internal context

### Structural characteristics

AGIR and the monitoring committee are the two structures that were in charge of implementation. Their mode of structuring and functioning was considered as elements that negatively influenced the implementation. The study participants concluded that the monitoring committee did not function well, which did not allow it to provide the expected facilitation support during the operationalization of the intervention. They felt that the people in charge of AGIR were involved in the activities. The lethargy of this body has negatively influenced the implementation of activities through weak monitoring, a lack of support for mobilization. Field participants in two of the three sub-areas noted this deficiency.

*“Throughout the execution of the activities, I did not hear a facilitator from our group say that the committee members supported him. They didn't participate in anything. What did this instance even serve?” (Facilitator, Yitouni Zone).*

### Network and communication

Study participants felt that the working atmosphere was cordial with a regular exchange between teams, sharing of challenges and successes. All the participants have demonstrated a good willingness to collaborate, to work with respect for others. This has created a pleasant atmosphere of collaboration and commitment from all. This has positively influenced the performance of the intervention team.

*“Everyone made the effort to open up to others and be understanding, which made the work easier because there was a lot of discussion to do a quality job” (Facilitator, Tampouy Bilbalogho Zone).*

### Preparation of implementation

The mobilization of health and political leaders in the area was weak, as was the involvement of leaders of community organizations. The team had difficulty mobilizing the population for advocacy activities. However, the context changed when a dengue epidemic hit the whole country in August 2016 [30]. It is at this time that both communities, political, administrative, and community authorities have shown sustained attention to the activities of the intervention through sincere involvement. This variation in the siting climate had a positive as well as a negative influence on the intervention. Indeed, before the outbreak of the dengue epidemic in Ouagadougou, many participants did not attach enough importance to intervention activities. When many people started contracting dengue fever, then they began to take part in the activities organized as part of the community-based dengue control intervention.

*“When we started implementing the activities, it was very difficult to mobilize local participants. Many did not know about this disease. Things have changed since the outbreak of a dengue epidemic in 2014. This situation subsequently facilitated the mobilization of the population and the authorities” (Member of the Monitoring Committee).*

### Commitment of the leaders

With regard to the commitment of leaders, the perceptions developed by participants are largely positive. Indeed, they considered that the managers of the structures involved in the implementation of the activities have shown a constant availability by fully playing the roles assigned to them. They noted their presence at the various meetings and their support of the teams in the field. The participants considered that the commitment of the leaders to positively contribute to motivate the participants on the ground and thus to influence qualitatively the actions deployed.

*“You know if you are involved in an action and you find that the first people in charge are not involved properly, you will eventually get discouraged. We noticed a real involvement of the leaders, which helped to mobilize the others” (Member of the monitoring committee)*

### Available resources

The quality of the training received on dengue fever, the knowledge acquired on animation and population outreach techniques, image boxes, and monitoring tools are the main resources that were made available to participants for the implementation of activities. They appreciated the quality of these resources and indicated that they enabled them to carry out the tasks entrusted to them without great difficulty. They especially stressed the relevance and richness of the information received during the various training sessions. The availability and quality of resources were perceived as participants that positively influenced the enthusiasm and quality of the work done.

## Characteristics of individuals

### Knowledge and beliefs about the intervention

The data collected from implementing participants indicate a perfect knowledge of the intervention. Indeed, both the facilitators and the members of the monitoring committee all cited the various elements of the project without any difficulty. They also used their participation as a way to strengthen their own knowledge about this dangerous disease, which is not well known to the general public, but also as an opportunity to be useful in serving the community.

“The outbreak of the dengue fever epidemic in the country has made us more proud of the work done and the good that has been done to many people through awareness” (Facilitator, Yitouni Zone).

### Self-efficacy

Study participants positively assessed their ability to implement the activities entrusted to them. They noted that the information made available to them and the mastery of the animation techniques learned were of high quality. With these means, they were able to carry out all the different tasks of the project as indicated. Mastery of animation techniques illustrated in the animation is one of the behaviors that required self-confidence on the part of the participants. The good perception of their intervention capacities positively influenced the implementation of the intervention.

## Process

### Planification

The participants adopted a participatory approach to activity planning. One facilitator explained:

“The timeline has always been a negotiated tool and is based on the availability of each other while taking into account the requirements of the implementation context.”

It has happened on several occasions that the established program has been modified due to constraints of availability of participants and the vagaries of time. Everything was organized in a concerted way. The participatory nature of the planning was well appreciated by the various stakeholders. They consider that this has contributed significantly to the effectiveness and efficiency of the organization of activities.

“If planning had been imposed, it was certain that we would not be able to exhaust the program of activities mainly because of the availability of people,” *(Member of the monitoring committee)*.

### Implication

Participants developed good perceptions about involvement. Indeed, they acknowledged that the process of implementing the intervention involved several types of participants. The promoters of the intervention took the time to meet as many political, health, and community participants as possible in the area, to explain the content of the intervention and the implementation strategy. As a result of this process, implementing partners were selected. One participant noted:

“This process made it possible to choose the people who wanted to work and not those who are only looking for money. And what has facilitated collaboration and seriousness in the work to achieve the results achieved.”

The involvement of the different categories of participants was considered effective by the participants, who considered that this strategy helped to facilitate the organization of activities by combining efforts.

### Formally appointed internal leaders for implementation

The individual and collective leadership fostered by a sharing of responsibilities among the various participants in the intervention has, according to them, had a positive influence on implementation.

“In the group of facilitators, we think that this way of working is better than some of the experiences we have had. In many interventions, that's not how things work. When you are a facilitator, we don't ask for your opinion. It is the people in charge who decide everything and you only follow. But here, it was the opposite, it was between us that we organized the activities without pressure. It was a good example” (Facilitator,).

### Champions

“We cannot say that this intervention has led to the emergence of other leaders because those who should be accompanying it have not” .

Many believe that an intervention of limited temporal and spatial scope cannot facilitate the emergence of new leaders. The intervention could not generate the emergence of people who were able to influence the behavior of other community members through their commitment.

Table 4 summarizes the empirical results.

**Table 4:** Summary of results by construct

Dimensions	Constructions	Data synthesis
1. Characteristics of the intervention	1.1 Origin of the intervention	It positively influenced intervention in all sub-areas.
	1.2 Quality and Strength of Evidence	The intervention was designed on the basis of evidence and community preferences.
	1.3 Adaptability	For two sub-areas, the intervention met needs; for one sub-area, it did not.
	1.4 Complexity	The intervention was simple to implement.
2. External Context	2.1 Network	AGIR did not have networks of relationships in the area.
3. Internal Context	3.1. Structural characteristics	The implementing structures did not have a good internal organization.
	3.2 Networks and communications	Communication between the participants involved in implementation has worked well.
	3.3 Preparation of implementation	The climate varied between the beginning (negative) and end of the intervention (positive).
	3.4 Commitment of the leaders	The people in charge of AGIR were well involved in the implementation.
	3.5 Available resources	Resources were judged by some to be insufficient and by others to be sufficient.
4. Characteristics of individuals	4.1 Knowledge and beliefs about the intervention	The intervention was consistent with the beliefs and expectations of the participants.
	4.2 Self-efficacy	Intervention participants engaged in activities differently.
5. Process	5.1 Planification	Overall business planning was satisfactory
	5.2 Implication	Participants were involved at all stages of the intervention.
	*5.3* Formally appointed internal leaders for implementation	The organizational mode resulted in the emergence of leaders within each team.
	5.4 Champions	The intervention was not able to generate new leaders.

## Discussion

Two dimensions emerge from the results as having had a positive influence on the implementation of this community-based dengue fever prevention intervention: (i) the characteristics of the intervention and (ii) the processes of the intervention implementation. On the other hand, the dimension of the external context had a negative influence on the implementation of the intervention.

The majority of the CFIR constructs were considered to have had a positive effect on implementation. Participants highlighted the need to consult local stakeholders to decide on the intervention and to implement activities that have already proved successful locally or in other contexts in order to obtain the best possible support from all stakeholders, including leaders. Having evidence is very important for health interventions. Indeed, they contribute to facilitating the mobilization of stakeholders during the implementation phase [31, 32]. The commitment of leaders is also one of the constructs that has a positive influence on implementation. The role of champions and leaders in the effectiveness of interventions has been highlighted in the health promotion literature for a very long time [31] as well as for the health system in Burkina Faso [33, 34].

Another set of constructs whose positive influence was revealed by participants are those of the complexity of the intervention and knowledge and beliefs about the intervention. The participants perceived the intervention as simple. Making an intervention simple is certainly an important element when we know that in the end, it is only an event in an already existing system that is complex by nature [35]. Participants had an excellent knowledge of the activities to be organized, and thus, their implementation was facilitated. In addition, the constructs of participatory “planning” and “internal leadership,” individual and collective, fostered by a sharing of responsibilities between the different participants of the intervention have positively influenced implementation. Collaborative planning is indeed a mechanism for effective community health interventions. The constructs that have most positively influenced the intervention are those relating to the involvement and participation of the intervention participants, as evidence about effectiveness studies on health promotion intervention show [36]. They were able to participate extensively in the reflection and implementation of the intervention. In Latin and Central America and South-East Asia, where dengue prevention interventions are more widespread, research shows that social participation is essential [37, 38]. In the same capital of Burkina Faso, one of our other studies showed that this social mobilization was not necessarily easy, showing the importance of the dynamics of the intervention, the context, and the participants in charge of it [39]. The only structure that has been judged negatively is the “network” due to the limited presence of the AGIR association on the ground. We know how essential the role of networks is to the implementation of community activities in Africa [40]. This shows the importance of relations with state, private, and associative structures in the intervention area and the need to establish a link prior to the implementation of a large-scale intervention. Vertical interventions without the participation of populations, often based solely on intra- or peri-housing spraying, have proven to be ineffective and unsustainable in containing mosquito expansion [41].

Many studies have demonstrated the effectiveness of interventions involving communities and even interventions that are fully initiated and supported by the communities themselves [7]. There is an added value of capacity building programs nestled in routine dengue fever prevention programs [42]. Although structured analyses of implementation remain very rare. To our knowledge, this is the first time that CFIR has been used in such a context of vector disease control in Africa. The lack of research on the implementation of this type of community intervention is obvious [43]. The fight against vectorborne diseases is a global public health problem, particularly with the impact of global warming, the increasing mobility of global populations, and urbanization. It is, therefore, urgent to consider the conditions for implementing interventions in order to improve the transferability of effective interventions from one context to another.

Although some authors may criticize its overly generic nature [44], its ease of adaptation to a particular context in Burkina Faso has been important, like it has been done elsewhere in Africa [45]. Recent research in Burkina Faso has shown that an adaptation of the CFIR to context could be fruitful to explain the factors that influence implementation [46]. Indeed, the CFIR is often used to analyze the implementation processes of knowledge-transfer interventions [47]. The systematic nature of its implementation makes it possible to cover a wide range of participants that have potentially influenced the implementation of our intervention. The discussions generated during the workshops also help to understand how these implementation participants can influence the performance of our intervention. Like other authors [48], the use of a measurement scale for the CFIR constructs by participants to guide, without measuring, the continuation of qualitative data collection has been fruitful. *Aedes* control interventions are limited to quantitative impact analyses when we have much to learn from the underlying processes. Qualitative analysis is, then, essential and is still lacking [43]. This study helped to understand the importance of the influence of contextual participants on an intervention. The key is not to just design an intervention properly and mobilize financial resources, but also to put in place appropriate implementation strategies that take into account the intervention environment. The relevance of this research lies in the fact that it could inspire new studies on the challenges of implementing interventions in the African context. This issue is crucial to the success of development policies.The recent attempt has been made by researchers to create a CFIR Inner Setting scale [8].

Methodological limitations are related to the fact that we were not able to use statistical data and in-depth analyses to quantify certain dimensions of the CFIR. Furthermore, this is not a completely objective or distanced analysis of the intervention, as some of the authors of this article were involved in defining and monitoring the intervention. This conflict of interest can be a limitation, but can also be understood as a strength of the research because it allows an in-depth analysis of the context and the stakes of the intervention. The first author was not involved in the initial research protocol or in the design and implementation of the intervention. In addition, the analysis was rigorous and the process was transparent. It was supported by a recognized conceptual framework.

The practical implications of this study relate to the need to conduct studies of community preferences and local contexts in order to define the content and the design of interventions. In particular, it is important to have a good understanding of local issues and the community leaders to be mobilized in order to adapt the content of interventions and especially their implementation specifically to local contexts. From a scientific standpoint, we have shown not only the relevance of using the CFIR, but also its challenges and the need to adapt it to research needs. It is essential not only that researchers take the time to document these challenges and publish their reflective analysis but also that scientific journals may be able to give this space for reflection. There is an urgent need to continue to develop and implement community-based dengue control interventions, to evaluate their effectiveness, and, most importantly, to understand their processes with social science experts and social science theories.

## Conclusion

The study achieved its objectives by identifying the elements of context that positively influenced implementation and those that constituted barriers. The results of the study indicated that the origin of intervention, quality and strength of evidence, self-efficacy, and planification made the intervention easier. However, network, available resources, and structural characteristics have negatively influenced the implementation of a community-based intervention to control dengue fever in Burkina Faso.

## Data Availability

The dataset (which includes individual transcripts) is not publicly available due to confidentiality policies.

## Abbreviations

AGIR/SD: Action, Gouvernance, Intégration et Renforcement en Santé et Développement
CFIR: Consolidated Framework for Implementation Research
